# The Mediating Effects of Burnout on the Relationship between Anxiety Symptoms and Occupational Stress among Community Healthcare Workers in China: A Cross-Sectional Study

**DOI:** 10.1371/journal.pone.0107130

**Published:** 2014-09-11

**Authors:** Yanwei Ding, Jianwei Qu, Xiaosong Yu, Shuang Wang

**Affiliations:** Department of General Practice, The First Affiliated Hospital, China Medical University, Shenyang, Liaoning, China; University of Stellenbosch, South Africa

## Abstract

**Background:**

Several occupational stress studies of healthcare workers have predicted a high prevalence of anxiety symptoms, which can affect their quality of life and the care that they provide. However, few studies have been conducted among community healthcare workers in China. We attempted to explore whether burnout mediates the association between occupational stress and anxiety symptoms.

**Methods:**

A cross-sectional survey was completed in Liaoning Province, China from November to December 2012. A total of 1,752 healthcare workers from 52 Community Health Centers participated in this study, and all participants were given self-administered questionnaires. These questionnaires addressed the following aspects: the Zung Self-Rating Anxiety Scale, the Chinese version of the effort-reward imbalance scale and the Maslach Burnout Inventory–General Survey. Finally, the study included 1,243 effective respondents (effective response rate, 70.95%). Hierarchical linear regression analysis, performed with SPSS 17.0, was used to estimate the effect of burnout.

**Results:**

The prevalence of anxiety symptoms among the community healthcare workers was 38.0%. After adjusting for demographic characteristics, the effort–reward ratio and overcommitment positively predicted anxiety symptoms. Meanwhile, the effort–reward ratio and overcommitment were positively related to the emotional exhaustion and cynicism subscales of burnout. In addition, the emotional exhaustion and cynicism subscales were positively related to anxiety symptoms. Thus, there is a link between burnout, occupational stress and anxiety symptoms.

**Conclusions:**

Burnout mediates the effect of occupational stress on anxiety symptoms. To effectively reduce the impact of occupational stress on anxiety symptoms, burnout management should be considered.

## Introduction

With improved economic development, aging populations and lifestyle changes, an increasing number of chronic diseases seriously threaten population health in China. In such cases, more cost-effective, convenient primary care is particularly important. In the latest health care reform, China aimed to ensure equitable access to basic health care for its residents by building a strong, primary-care-based delivery system [1]. However, the Community Health Centers (CHCs) form the main pillar of primary-care providers in urban areas [2]. To maintain the desirability of primary healthcare, it is important to promote the physical and mental health of healthcare workers. However, few studies have investigated anxiety in CHC healthcare workers.

A previous study has shown that healthcare workers have a greater risk of developing anxiety (due to fear of making mistakes at work or impending weekends on duty), which is one of the most common psychiatric conditions encountered in primary care [3], [4]. Anxiety “is a psychologic and physiologic state characterized by cognitive, somatic, emotional and behavioral components” [5]. Anxiety is also described as a feeling of fear related to some uncertain or future event or mental distress that is caused by a threat to a person or his/her values [6]. A recent study reported that anxiety is associated with job burnout, sleeping problems and lifestyle factors [7]. Increasingly, data have indicated the adverse effects of anxiety. Anxiety can impair healthcare workers' quality of life, induce immune system changes [8], affect the safety of their patients and influence their job performance, which is reflected in both the greater risk of mistakes and the effects on their interactions with patients and colleagues [7], [9]. Healthcare worker anxiety undoubtedly has profound social effects. Many epidemiologic studies have suggested that psychiatric and non-psychiatric patients with chronic anxiety may be at risk for developing coronary heart disease [10]. To promote health in healthcare workers and to ensure that they provide high-quality care services, more attention should be directed toward reducing anxiety in healthcare workers.

Occupational stress has been identified as a risk factor for anxiety symptoms [7], [11]. Job demands, extrinsic effort, and over-commitment were associated with higher levels of anxiety [12]. Peplińska A et al. proposed that marital satisfaction mediated the relation between stress and anxiety [13]. According to the results of previous studies, occupational stress not only exerts a direct effect but also has an indirect effect on anxiety symptoms.

In particular, the effect of occupational stress on physical and mental illness has attracted attention [14]. Occupational or work-related stress is undoubtedly a major cause of mental health concerns worldwide [15]. However, healthcare workers, including physicians, nurses, medical technicians and administrative staff, experience high levels of occupational stress because of their heavy workloads, extended working hours and time-related pressure [16]. Additionally, Chinese healthcare workers are likely more vulnerable to increasing occupational stress because of the great demand for service quality, hospital-patient conflicts and health care reform. The mechanism by which occupational stress affects anxiety is worth exploring to provide efficient strategies for anxiety intervention.

Burnout is a well-known psychological response in healthcare workers [17]. In addition, burnout is consistently associated with anxiety symptoms [18]–[20]. Attention should be focused on preventing exhaustion from work with the purpose of lessening anxiety symptoms [7]. The outcome of continued exposure to overwork, resulting in a progressive inability to perform job responsibilities, has been termed ‘burnout’ [8]. Burnout is characterized by emotional exhaustion (feeling emotionally overloaded with work), cynicism (active disengagement from one's job) and inefficacy (decreased personal accomplishment) [21]. Burnout is a prolonged response to chronic job-related stressors; therefore, it has a special significance in health care, in which staff experience both emotional and physical stresses [22]. High burnout scores are usually associated with the following variables: low salary, employment sector, age group, feeling of professional inadequacy and feelings of personal dissatisfaction [23]. Several studies have shown that occupational stress has significant power in predicting burnout [24]–[26], particularly in the emotional exhaustion and cynicism dimensions.

To the best of our knowledge, the effect of occupational stress on anxiety symptoms has been tested, but few studies have addressed the potential mechanism underlying the relationship between occupational stress and anxiety symptoms. Moreover, Gilbert et al. reported that burnout mediates the relationship between structural empowerment and organizational citizenship behaviors [27]. However, whether burnout mediates the relationship between occupational stress and anxiety symptoms in healthcare workers who mainly provide primary care in CHCs has not been investigated. To explore the mechanism between occupational stress and anxiety symptoms, it is important to understand the role of burnout in this relationship.

In this study, we investigated the prevalence of anxiety symptoms in community healthcare workers in China and hypothesized that burnout mediates the relationship between occupational stress and anxiety symptoms.

## Methods

### Ethics Statement

The study procedures were approved by the relevant ethical standards of the Committee on Human Experimentation of China Medical University. Written informed consent was obtained from each participant, all of whom voluntarily participated in the study. We protected the privacy and anonymity of individuals involved in our research.

### Study Population and Procedure

A cross-sectional survey was conducted in Liaoning Province, China from November to December 2012. Participants in this study were selected using multi-stages cluster sampling. In the first stage, three cities at each financial level (GDP<100 billion, GDP = 100–200 billion and GDP>200 billion) were randomly selected based on the economic development of Liaoning Province. In the second stage, we randomly chose municipal districts from the 1/2 ratio from each selected city. In the third stage, three CHCs were randomly chosen from each selected municipal district. All healthcare workers in each CHC were invited to participate in our study. In total, 1752 healthcare workers were selected from fifty-two CHCs in nine cities. After all participants provided written informed consent, they received a self-administered questionnaire. We received effective responses from 1243 individuals (effective response rate, 70.95%), who formed the study sample. The individuals in this manuscript provided written informed consent (as outlined in the PLOS consent form) to publish these case details.

### Measurement of Anxiety Symptoms

The Zung Self-Rating Anxiety Scale (SAS) was used to measure the anxiety related symptoms of the healthcare workers. This scale was prepared by Zung in 1971 [28] to evaluate the severity of anxiety symptoms in the investigated objects; it consists of 20 items. Each item is scored on a scale of 1–4 (never or occasionally, sometimes, frequently and most of the time), and the final score ranges from 20 to 80. In accordance with the Chinese norm, we defined the anxiety symptoms for a total raw score ≥40 [29]. Additionally, a higher score represented more serious anxiety symptoms.

The Chinese version of the questionnaire has previously been used in Chinese populations and is reliable [30], [31]. In the present study, Cronbach's alpha in SAS was 0.85. After revising several items, the confirmatory factory analysis confirmed the validity of the scale (RMSEA = 0.071, CFI = 0.902, GFI = 0.918).

### Measurement of Occupational Stress

The effort-reward imbalance (ERI) model was first proposed by Siegrist in 1996 [32] to explain the psychological well-being in the workplace. This model emphasizes the mismatch between the high effort that individuals exert and the low reward they receive in occupational life, in which rewards are provided as money, esteem and career opportunities, including job security [33], [34].

The Chinese version of the ERI questionnaire, translated by Li and Yang, was used in this study [35]. The scale consisted of the following three dimensions: effort (6 items), reward (11 items) and overcommitment (6 items). The effort and reward dimensions represent extrinsic stress, and overcommitment represents intrinsic stress. For the effort and reward subscale, each item is scored from 1 to 5; 1 indicates the lack of a particularly stressful experience, and 5 indicates a high-stress experience. Participants answered the items in two steps. First, they indicated whether they agreed or disagreed with the item's description of their work situation; then, they were asked to evaluate how distressed they feel (from ‘not distressed’ to ‘very distressed’). The effort–reward ratio (ERR) is calculated with a predefined algorithm [36] and is calculated by dividing the effort score by the reward score, for which the latter score is multiplied by a correction factor of 6/11. For the overcommitment subscale, each item is scored from 1 (full disagreement) to 4 (full agreement).

The Chinese version of the ERI questionnaire is a reliable, valid instrument for measuring psychosocial stress at work, particularly among Chinese healthcare workers [35]. Cronbach's alpha for the effort, reward and overcommitment are reported as 0.77, 0.81 and 0.66, respectively [37]. In our study, Cronbach's alpha for the effort, reward and overcommitment was 0.85, 0.89 and 0.71, respectively. After revising seven items, the confirmatory factory analysis confirmed that the Chinese version of the ERI had a satisfactory goodness-of-fit (RMSEA = 0.057, CFI = 0.923, GFI = 0.925).

### Measurement of Burnout

Burnout was measured by the Maslach Burnout Inventory-General Survey (MBI-GS) [38], which includes the following three dimensions: emotional exhaustion (EE), cynicism (CY) and professional efficacy (PE). The total scale consists of 15 items, with five items measuring the EE subscale, four items measuring the CY subscale and six items measuring the PE subscale. Additionally, each item is rated from 0 (never) to 6 (every day) according to how often the statement is experienced [39]. A greater degree of burnout is predicted by higher scores for the emotion exhaustion and cynicism subscales and by lower scores for the professional efficacy subscale.

The Chinese version of the BMI-GS has high validity and reliability among Chinese medical professionals [40]. In our study, Cronbach's alpha was 0.84 for the BMI-GS and 0.94, 0.92 and 0.93 for the EE, CY and PE subscales, respectively. After revising ten items, the confirmatory factory analysis confirmed that the Chinese version of the BMI-GS had a satisfactory goodness-of-fit (RMSEA = 0.079, CFI = 0.963, GFI = 0.929).

### Demographic Characteristics

The length of employment (years), weekly working hours and marital status were obtained in this study. Marital status was categorized as single, married/cohabitation and divorced/ separated/widow. The length of employment (working years) was categorized as ≤10 years, 11–20 years, 21–30 years and >30 years. The weekly working hours was categorized as ≤40 hours and >40 hours.

### Statistical Analysis

The study variables were compared among the marital status groups, length of employment groups and weekly working days groups by an independent-samples T-test and one-way analysis of variance (ANOVA). Pearson's correlation coefficients were used to examine the correlations among the study variables. Multiple regression analysis using the hierarchical stepwise method was applied to examine whether burnout played a mediating role in the association between occupational stress and anxiety symptoms, according to the criteria proposed by Baron and Kenny [41]. In the regression equation, occupational stress was modeled as the independent variable, anxiety symptoms as the dependent variable, burnout as the mediator, and length of employment as the control variable. According to Baron and Kenny, for burnout to be a mediator between occupational stress and anxiety symptoms, the following four conditions should be met: (1) occupational stress significantly predicts anxiety symptoms; (2) occupational stress significantly predicts burnout; (3) burnout significantly predicts anxiety symptoms; and (4) the relationship between occupational stress and anxiety symptoms is reduced or insignificant upon the addition of burnout.

Before the regression analysis, we measured tolerance and variance to exclude multicollinearity. The independent variables were entered in the hierarchical linear regression equation in three steps. In the first step, the length of employment was added as the control variable; according to one-way ANOVA analysis, the only difference in the anxiety symptoms was for the length of employment. All significant occupational stress variables, including ERR and overcommitment, were included in the second step. All significant burnout variables, such as EE, CY and PE, were included in the third step. Additionally, we performed the Sobel test to determine the mediation role of burnout [42]. Except for the confirmatory factor analysis, all analyses were performed using SPSS 17.0 for Windows, and statistical significance was defined as p<0.05 (two-tailed). Confirmatory factor analysis was performed using Amos 17.0.

## Results

### Characteristics of the Study Population and the Prevalence of Anxiety Symptoms

The length of employment averaged 18.2 years (standard deviation [SD] 10.8), with 32.2% (400) of the participants working more than 40 hours per week; 79.3% (985) were married or cohabiting. The mean anxiety symptom score among the participants in the present study was 37.7 (SD 9.7), and the prevalence of anxiety symptoms was 38.0%.

There were significant differences in all study variables for the length of employment (p<0.01). The EE (p<0.05) and CY (p<0.001) scores differed across weekly working hours. However, no significant difference was observed in the weekly working hours groups with respect to ERR, overcommitment, PE and anxiety symptoms. Within each marital status group, there was only a significant difference in the score for overcommitment. The participant characteristics and distributions of anxiety symptoms for the categorical variables are shown in Table 1.

**Table 1:** Participant characteristics and the distributions of anxiety symptoms by categorical variable.

Category	Subcategory	Result (%)	Anxiety symptoms
			Mean (SD)
Length of employment	≤10 yr	357 (28.7)	39.1 (10.0)
	11–20 yr	390 (31.4)	37.3 (9.5)
	21–30 yr	328 (26.4)	37.4 (9.3)
	>30 yr	168 (13.5)	35.9 (9.9)
F			4.834
p			0.002
Weekly working hours	≤40 hr	843 (67.8)	37.5 (9.5)
	>40 hr	400 (32.2)	38.1 (10.2)
t			−1.040
p			0.299
Marital status	single	188 (15.1)	37.9 (9.8)
	married/cohabitation	985 (79.3)	37.6 (9.7)
	divorced/separated/widow	70 (5.6)	38.0 (10.1)
F			0.093
p			0.911

### The Relationship Between Occupational Stress, Burnout and Anxiety Symptoms

Correlations between the study variables are shown in Table 2. Length of employment was negatively related to ERR, EE, CY and anxiety symptoms. ERR, overcommitment, EE and CY were positively correlated with anxiety symptoms, whereas the length of employment and PE were negatively correlated with anxiety symptoms. ERR had a significant, positive relationship with EE and CY, and overcommitment had a significant, positive correlation with all three burnout dimensions.

**Table 2:** Means, standard deviations (SD) and correlations of all variables.

	Mean (SD)	1	2	3	4	5	6
1. Length of employment	18.2 (10.8)						
Occupational stress							
2. Effort-reward ratio	0.8 (0.3)	−0.08**					
3. Over commitment	15.5 (3.0)	0.11**	0.27**				
Burnout							
4. Emotional exhaustion	10.1 (6.5)	−0.10**	0.49**	0.39**			
5. Cynicism	5.7 (5.2)	−0.14**	0.36**	0.16**	0.60**		
6. Professional efficacy	24.1 (9.3)	0.08**	−0.06*	0.06**	0.01	−0.21**	
Anxiety							
7. Anxiety symptoms	37.7 (9.7)	−0.10**	0.34**	0.18**	0.46**	0.49**	−0.26**

*p<0.05, **p<0.01 (two-tailed).

### Burnout as a Mediator of the Relationship between Occupational Stress and Anxiety Symptoms

**Figure 1:**
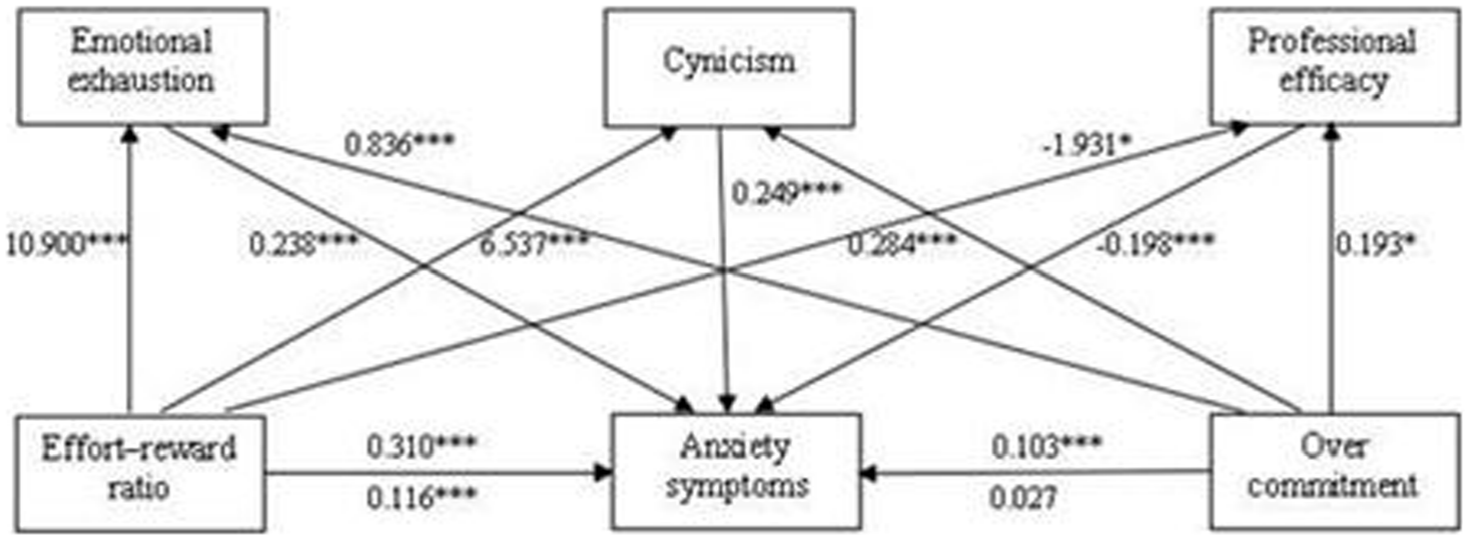
The mediating model: relationships between occupational stress and anxiety symptoms. β–coefficient above the line: without the mediation; β–coefficient below the line: with the mediation. * p<0.05, *** p<0.001.

The results of the hierarchical linear regression analysis are shown in Table 3. The control variable (length of employment) significantly predicted anxiety symptoms in the first step (R^2^ = 0.009). ERR and overcommitment were significant predictors of anxiety symptoms in the second step and accounted for 12.3% of the variance. In the third step, EE and CY positively predicted anxiety symptoms, whereas PE negatively predicted anxiety symptoms, which explained an additional 19.3% of the variance. When EE, CY and PE were included, the relationship between ERR and anxiety symptoms was significantly reduced (from β = 0.310 to β = 0.116, p<0.001; Sobel test, z = 12.05, p<0.001), whereas overcommitment failed to reach the level of significance (from β = 0.103 to β = 0.027, p>0.05; Sobel test, z = 7.68, p<0.001). Therefore, burnout mediated the effect of occupational stress on anxiety symptoms (depicted in Figure 1).

**Table 3:** Hierarchical linear regression analysis results.

	Anxiety symptoms		
	Step 1	Step 2	Step 3
Length of employment	−0.097**	−0.085**	−0.019
Effort–reward ratio		0.310***	0.116***
Over commitment		0.103***	0.027
Emotional exhaustion			0.238***
Cynicism			0.249***
Professional efficacy			−0.198***
R^2^	0.009	0.133	0.326
ΔR^2^	0.009**	0.123***	0.193***

*p<0.05, ** p<0.01, *** p<0.001 (two-tailed).

## Discussion

The subjects in this study were selected from Liaoning Province using multistage cluster sampling. The subjects seemed to be representative of our study population, allowing for generalization of our conclusions. We examined the prevalence of anxiety symptoms and explored the relationship between occupational stress and anxiety symptoms in community healthcare workers in China. In total, 38.0% of the healthcare workers had anxiety symptoms. The prevalence was more than twice the prevalence in Chinese medical students (12.5%) [28] and the general population in Australia [43], as well as the prevalence for the elderly general population reported by Schwarz et al. [44].

The level of stress in healthcare workers has attracted increasing attention because of concern for the health of the individuals themselves and because of the possible impact on the quality of patient care [45]. Several studies have shown that healthcare workers, particularly nurses, have a greater risk than that of other professionals of developing emotional distress from job stress, such as burnout, anxiety and depression [46]. Rapid economic development, medical reform, aging populations, a shortage of qualified personnel and the growing burden of chronic diseases increase the job demands on community healthcare workers who are expected to meet their patients' basic health needs through effective, economic, comprehensive and continuous services [1], [47], [48]. Thus, in addition to their default challenges, healthcare providers have to balance the expectations of funding agencies, administrators and patients. This result is further complicated by the geographical inequity of health resources, fierce competition over work positions, problematic scheduling associated with shiftwork, emotional issues from handling illness or death, working relationships, demand for providing better medical services and increasingly complex doctor-patient relationship [11], [48]–[50], all of which increase a healthcare provider's workload. Of course, the challenges with which they are faced are not limited to those described above. The reward these workers receive is usually insufficient because of their low salaries. When intense effort is not met with adequate rewards, anxiety is more likely to develop.

The association between occupational stress and anxiety symptoms was consistent with a previously published study [51], in which both the positive effect of the extrinsic stress (ERR), and the effect of intrinsic stress (overcommitment) on anxiety symptoms was significant. Reasonable financial compensation and other effective strategies should be provided to balance the workload and reward. A possible explanation for the significance of overcommitment is that 32.2% of healthcare workers work more than 40 hours per week, and work is a significant part of their daily life. These healthcare workers do not have sufficient personal lives, which would otherwise relieve their stress.

In this study, we examined the relationship between the dimensions of burnout and anxiety symptoms. Emotional exhaustion and cynicism were positively related to anxiety symptoms, and professional efficacy was negatively related to anxiety symptoms. These results were correspondent with the results of a previous study [7]. Among Chinese healthcare workers, feeling emotionally overloaded with work or active disengagement from their work seems to make individuals prone to anxiety symptoms. They would fear making mistakes or impending weekends on duty.

Moreover, we found that length of employment was negatively related to ERR, emotional exhaustion, cynicism and anxiety symptoms. One possible reason for the negative relationship between length of employment and ERR is that the longer healthcare workers work, the more reward they achieve, such as a raise in salary. In this case, we can explain the relationship between length of employment and emotional exhaustion, cynicism and anxiety symptoms by the effect of occupational stress on burnout and anxiety symptoms.

As previously mentioned, more attention should be given to reducing occupational stress and alleviating anxiety symptoms in healthcare workers. We attempted to explore the mechanism by which occupational stress affects anxiety symptoms. We hope our results can help to develop effective interventions for anxiety.

This study is the first to verify that burnout mediates the association between occupational stress and anxiety symptoms in community healthcare workers in China. Apart from the direct effect of occupational stress on anxiety symptoms, the results of this study indicated that through the mediation of burnout, occupational stress could also affect anxiety symptoms. High occupational stress may enhance burnout, thereby causing anxiety. Health managers should be aware of the prevalence of emotional exhaustion and cynicism in the workplace to ensure that they develop strategies to advance professional efficacy and to improve the health status of healthcare works. Gómez-Gascón et al. researched interventions for preventing and treating burnout in primary health care professionals [52]; the authors reported that organizational measures are important for preventing burnout syndrome. Additionally, healthcare professionals should be provided with coping strategies. Another study in Canada showed that psychological empowerment in healthcare workers can reduce the effects of stressors on burnout [47].

This study has some limitations. First, we conducted a cross-sectional investigation; therefore, we cannot define causal relationship between occupational stress, burnout and anxiety symptoms. These findings should be confirmed with longitudinal studies. Second, all data were based on self-reported questionnaires; therefore, bias introduced by the common method variance cannot be avoided. Moreover, healthcare workers belong to a special group, and we cannot extended the results to other populations without clear evidence.

## Conclusions

There was a high prevalence of anxiety symptoms in community healthcare workers in China. After adjusting for demographic characteristics, the extrinsic stress (ERR), intrinsic stress (overcommitment), emotional exhaustion and cynicism subscales were positively associated with anxiety symptoms. However, professional efficacy was negatively associated with anxiety symptoms. Additionally, burnout mediated the relationship between occupational stress and anxiety symptoms. The combination of decreasing occupational stress with reducing burnout may be more useful in preventing anxiety symptoms in Chinese Community Health Center healthcare workers.
